# Effect of smartphone-assisted lifestyle intervention in MASLD patients: a randomized controlled trial

**DOI:** 10.1038/s41598-024-64988-4

**Published:** 2024-06-17

**Authors:** Apichat Kaewdech, Suraphon Assawasuwannakit, Chaitong Churuangsuk, Naichaya Chamroonkul, Pimsiri Sripongpun

**Affiliations:** 1 Gastroenterology and Hepatology Unit, Division of Internal Medicine, Faculty of Medicine, Prince of Songkla University, Hat Yai, Songkhla 90110 Thailand; 2 Department of Medicine, Panyananthaphikkhu Chonprathan Medical Center, Srinakharinwirot University, Nonthaburi, 11120 Thailand; 3 Clinical Nutrition and Obesity Medicine Unit, Division of Internal Medicine, Faculty of Medicine, Prince of Songkla University, Hat Yai, Songkhla 90110 Thailand

**Keywords:** Metabolic diseases, Metabolic dysfunction-associated steatotic liver disease, Diet, Lifestyle, Smartphone, Hepatology, Hepatitis, Liver, Liver diseases

## Abstract

Metabolic dysfunction-associated steatotic liver disease (MASLD) is emerging globally as a significant problem. The mainstay of treatment is lifestyle intervention (LSI). We hypothesized that providing information regarding LSI and MASLD through a social media application generally used in the respective society would improve clinical outcomes in MASLD more than standard of care (SOC). This is a randomized controlled study in noncirrhotic MASLD patients aged 18–65 years in Thailand. Eligible patients were randomly assigned to either the control (SOC) or intervention arm. Patients in both groups received standard LSI advice. Infographics about MASLD and LSI information were sent to the intervention group every 3–7 days via the LINE official account. The outcomes are changes in liver steatosis and liver stiffness by FIBROSCAN at 24 weeks, as well as weight loss, body composition, and serum alanine aminotransferase (ALT) level between the two groups. A total of 122 patients were enrolled. The median age of eligible participants was 53 years, 64.7% were female, and median body mass index was 27.3 kg/m^2^. After a complete 24-week study period, both groups had an improvement in weight, ALT level, liver steatosis, and fat mass, but the differences in those changes between groups were not statistically significant. Interestingly, a significant improvement in liver stiffness was observed in the intervention group than in the control group (− 0.7 ± 1.8 kPa vs. 0.1 ± 2.4 kPa, *P* = 0.035). Encouraging LSI and delivering MASLD information via a social media application (LINE official account) to patients with MASLD demonstrated a better outcome of liver stiffness measurement than SOC.

*Clinical trial number*: TCTR20210304002 (04/03/2021) (http://www.thaiclinicaltrials.org/show/TCTR20210304002).

## Introduction

Steatotic liver disease (SLD) becomes one of the major problems of serious clinical concern worldwide, as it is highly prevalent and can progress to liver cirrhosis and hepatocellular carcinoma^1–4^. Data from Wong et al.^5^ showed that the number of liver transplants due to hepatocellular carcinoma from fatty liver without significant alcohol consumption between 2002 and 2012 increased by nearly fourfold and became the third leading indication of liver transplantation in the United States.

Non-alcoholic fatty liver disease (NAFLD) is a term that has been used for a long time, defined as evidence of hepatic steatosis in patients without significant alcohol consumption. In 2020, a new terminology was proposed by an international consensus, namely metabolic dysfunction-associated fatty liver disease (MAFLD)^6,7^. More recently, in 2023, the new nomenclature with the overarching term of SLD comes with the new subclassifications of metabolic dysfunction-associated fatty liver disease (MASLD) substituted for NAFLD and endorsed by the international liver societies^8–10^. MASLD is a condition of liver disease characterized by excessive accumulation of fat in the liver, accompanied by at least one of five cardiometabolic risk factors.

Control of metabolic comorbidities and lifestyle intervention (LSI) are fundamental and the most important interventions in the treatment of patients with MASLD^11–13^. Yet only a few patients achieved the desirable weight loss goal^11^. Nowadays, technologies have an influence on daily living and become one of the important key factors in improving LSI, especially in patients with diabetes and obesity^12^. Either programs from internet websites or applications on mobile phones can be used for programming to promote lifestyle change, such as dietary control and exercise. We conducted this study aiming to determine the effect of LSI enhanced by LINE Official Account (using LINE mobile application) on liver steatosis, liver stiffness, and other associated parameters in MASLD patients compared to the standard of care (SOC).

## Materials and methods

### Study design

A randomized, double-blinded, controlled trial in noncirrhotic NAFLD/MASLD patients was conducted at Gastroenterology and Hepatology Outpatients Department, Songklanagarind Hospital, a tertiary care university hospital in Southern Thailand, between March 2021 and July 2022. The study was approved by the office of human research ethics committee, Faculty of Medicine, Prince of Songkla University (REC: 63-479-14-1). The study has already been registered with the Thai Clinical Trials Registry (TCTR), number TCTR20210304002. Informed consent was obtained from all participants. The study was conducted in accordance with the ethical guidelines of the 1975 Declaration of Helsinki.

### Study population

We enrolled patients aged between 18 and 65 who were diagnosed with MAFLD/MASLD. Patients with active malignancy, significant alcohol drinking (more than 21 standard drinks/week in men and more than 14 standard drinks/week in women), current pioglitazone or GLP1 agonist treatment, coexisting other liver diseases e.g., viral hepatitis or autoimmune hepatitis, patients with cirrhosis (fibrosis stage F4 by liver biopsy or liver stiffness > 17 kPa by FIBROSCAN^13^), pregnancy, unstable cardiovascular or neurological conditions, and those who are unwilling to participate were excluded.

All participants were randomly assigned into two groups, an intervention group and a control (SOC) group using block-of-four randomization and stratified by body mass index (BMI) [≥ 23 or < 23 kg/m^2^], and diabetes status. The random sequences were generated by a computer and concealed in opaque envelopes until they were delivered to the designated participants by SA, who was not involved in the study’s assessment.

### Study intervention and LINE application

After enrollment, all patients received MASLD (clinical importance of the disease and possible consequences) and LSI information (dietary, physical activity, and exercise advice) from a single hepatologist (AK). A standard video clip was introduced to all eligible patients; this process was considered to be SOC. Subsequently, each patient will be added to the LINE official account group separately for the intervention group and the control group. The patients and the doctors who followed up the patients (AK and PS) were blinded to their assigned group as the invitations to each LINE official account group were provided using different QR codes. The name and the profile picture for each LINE official account between the intervention and the control group were almost identical to one another.

LINE is the most commonly used social media application for chat and messaging in Thailand (98.5%)^14^. There were at least 44 million active users on the LINE application in Thailand as of 2019^15^, in which the overall Thai population of all ages was estimated at 69 million people at that time. And not only among teenagers and adults, LINE has been reported to be easy to use in the elderly in Thailand as well^16^. Therefore, we opted to use the feature of LINE official account from the LINE application as a tool to deliver MASLD and LSI information to the patients, as well as being a tool for reminding patients to perform LSI in Thailand.

The LINE official account has the feature of broadcasting messages and video clips to a target group of users (the users who were added to that LINE official account are being called followers, henceforth) in addition to the normal messaging function between the followers and the administrator of that account, while maintaining privacy among users. As the administrator will be the only person who is able to see all followers, the followers will not know who other followers are, and any inputs sent back from the followers will be only seen by the administrator, not the whole group of followers. This feature is an additional feature only for the LINE official account, unlike the usual LINE group, in which all members can see and chat with each other.

In the intervention group, one of the investigators (SA) was responsible for broadcasting knowledge about diet, physical activity, and exercise in MASLD to the patients periodically, as well as reminding them to do the LSI every 3–7 days. The video clips, infographics, or text content to be broadcasted to the patients in this group were curated and created by investigators, in which the content validation for the correctness of the literacy was assessed by the hepatology specialists of the Gastroenterology and Hepatology Unit, Faculty of Medicine, Prince of Songkla University. This content adhered to the recommendations on lifestyle modification for fatty liver patients from the American Association for the Study of Liver Diseases (AASLD), the European Association for the Study of the Liver (EASL), and the Asian Pacific Association for the Study of the Liver (APASL)^17–19^, as well as obesity management recommendations from the Royal College of Physicians of Thailand (RCPT)^20^. Examples of the content shared via the LINE official account in this group are presented in Supplementary Fig. 1.

In the control group (SOC), there were only the video clips they had watched on the date of enrollment in their LINE official account to rewatch. Neither the LSI broadcast nor the reminder were sent to this group. Prior to the study and during study visits, we instructed the patients not to discuss the information they received via the LINE application among themselves. All patients were followed up at 12 and 24 weeks after enrollment.

### Data collection

#### Clinical, laboratory parameters

Clinical and laboratory data were obtained at enrollment (baseline), 12-week and 24-week of follow up. Demographic data, such as age, sex, weight, height, waist circumference, and routine laboratory data, including complete blood count (CBC), blood urea nitrogen (BUN), creatinine (Cr), liver function test (LFT), gamma-glutamyl transferase (GGT), and lipid profiles were obtained in every visit. In addition to routine laboratory data, fasting blood sugar (FBS), hemoglobin A1C (HbA1C) and insulin levels were collected at baseline and at 24-week of follow up. The homeostasis model assessment parameter of insulin resistance (HOMA-IR) was calculated by the formula: [FBS (mg/dL) × fasting insulin (μU/mL)]/405.

#### Liver steatosis and liver stiffness measurement

Transient elastography was used to determine liver-specific outcomes in this current study. Quantitative measurement of the degree of liver steatosis was evaluated by the controlled attenuated parameter (CAP), and the degree of fibrosis was evaluated by liver stiffness measurement. CAP and liver stiffness measurements at enrollment (baseline) and 24-week follow-up were performed using a FIBROSCAN (Echosens FIBROSCAN 502 Touch). The M probe was used for participants with a BMI < 30 kg/m^2^, and the XL probe was used for those with BMI of 30 kg/m^2^ or higher. At least 10 valid measurements of liver stiffness data were accepted. All FIBROSCAN was performed by a single hepatologist (AK), who was blinded the assigned group of patients. AK received certified training from Echosens and had experience performing liver stiffness measurement using a FIBROSCAN 502 Touch for about 1,000 procedures.

#### Hand grip strength (HGS) test

HGS was measured at enrollment (baseline) and 24-week follow-up using a Jamar dynamometer. A maximum squeeze of at least 2 s for 3 attempts with the patient’s dominant hand was obtained. The average results of HGS were used. The strength measurement was performed in a 90-degree elbow flexed position.

#### Bioelectrical impedance analysis (BIA)

An eight electrode configuration portable BIA device (Tanita MC780 MA, Tokyo, Japan) was applied at enrollment (baseline) and 24-week follow-up. The fat and muscle mass were automatically calculated by the device. The patients were advised to remove the metallic objects and empty the bladder before the measurement. The details of the measurement were described previously^21^.

#### Outcomes

The main outcome was the change in liver steatosis (measured by CAP) between the intervention group and the control group. The secondary outcomes were the change in degree of liver fibrosis (measured by liver stiffness), as well as the changes in weight, body composition parameters, waist circumference, HGS, and serum alanine aminotransferase (ALT) level between both groups. Physical activity at baseline and at the end of the study was assessed using the WHO Global Physical Activity Questionnaire (GPAQ)^22^.

### Sample size calculation

From the study by O’Gorman et al.^23^, the investigators studied CAP in NAFLD patients between the exercise training group and the control group for 6 months. The CAP values of the intervention group and the control group were 290 ± 73 dB/m and 334 ± 36 dB/m at the end of the study, respectively. As our study did not provide a full exercise training session but delivered exercise and dietary knowledge and encouraged lifestyle modification. We assumed that the CAP in the intervention group in our study might not be as low as 290 dB/m; thus, we hypothesized that the predicted CAP in the intervention group would be 303 ± 73 dB/m instead of 290 ± 73 dB/m. Therefore, the numbers entered into the formula were as follows: mean in a treatment group = 303.00, SD. in a treatment group = 73.00, and mean in a control group = 334.00, SD. in a control group = 36.00, with a 1:1 ratio. One hundred and ten participants (55 participants in each arm) would be sufficient for the study with 80% power at the statistical level of 0.05, and assuming 10% loss to follow-up during the study period, a total of 121 eligible patients would be included.

### Statistical analysis

All statistical analyses were performed using R program version 4.2.3 (Vienna, Austria). Descriptive statistics were used to describe baseline characteristics. For continuous variables, the mean and standard deviation (SD) or median and interquartile range (IQR) were presented according to the distribution of the data. Vigorous and moderate intensity exercise time per week were retrieved from GPAQ question P10-15 and calculated according to the WHO GPAQ analysis guide^24^. Comparisons between 24-week follow-up and baseline in the same individuals were analyzed by a paired *t*-test or Wilcoxon sign-rank test according to the distribution of the data. To compare the outcomes between the intervention and the control group, both intention-to-treat and per-protocol analyses were carried out. The Chi-square test or Fisher Exact test for categorical variables and the Wilcoxon rank-sum test or t-test for continuous variables were used for the analyses as appropriate. A 2-tailed *p*-value of < 0.05 was considered to be statistically significant.

### Ethical approval

This research was conducted ethically in accordance with the World Medical Association Declaration of Helsinki.

## Results

### Baseline characteristics

**Figure 1:**
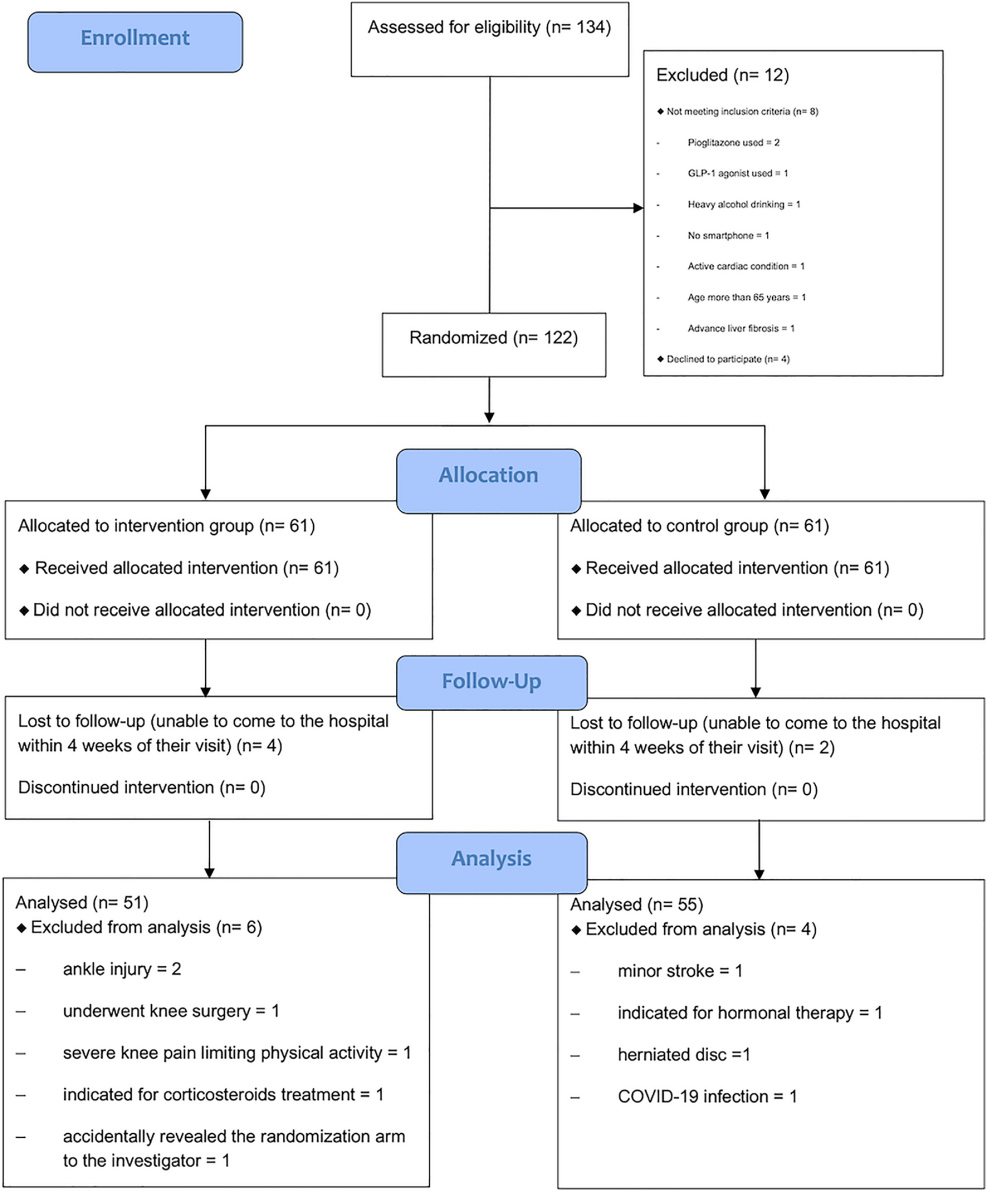
Patient’s flowchart presented the screening and randomization in this study.

During the study period, a total of 134 MASLD patients were screened. Of those, 122 participants were eligible and included in the study (Fig. 1). All participants were randomly assigned to the intervention group (n = 61) and the control group (n = 61). Baseline characteristics of all participants are shown in Table 1. The median age of participants in both groups was similar. Most participants had a BMI of 23 kg/m^2^ or higher (113 of 122 participants: 92.62%). There were no significant differences in BMI or waist circumference between the two groups. The main underlying comorbidities of enrolled participants were dyslipidemia (n = 90), hypertension (n = 32), and diabetes (n = 17). As expected, the patients exercised minimally, far less than the weekly recommendation for healthy adults^6^. No significant difference in baseline laboratory results, CAP, liver stiffness, or fat mass were observed between the two groups.

**Table 1:** Baseline clinical characteristics between intervention and control group (n = 122).

Variables	Control group (n = 61)	Intervention group (n = 61)	*P* value
Demographic characteristic			
Age: median (IQR), years	52.9 (43.2, 59.7)	53.8 (46.6, 57.4)	0.903
Sex: male, n (%)	20 (32.8)	23 (37.7)	0.705
Weight: median (IQR), kg	72.5 (64.4, 80)	70 (63.6, 82)	0.864
BMI: median (IQR), kg/m^2^	27.1 (24.9, 29.3)	28 (24.8, 30.1)	0.782
Waist: mean (SD), cm	93.3 (8.6)	92.8 (10.2)	0.748
Hand grip strength: median (IQR), kg	28.5 (26, 35.7)	27.7 (23.3, 35.3)	0.399
Moderate exercise activity, min/week	20 (0, 120)	45 (0, 90)	0.957
Vigorous exercise activity, min/week	0	0	0.191
Underlying comorbidities, n (%)			
Diabetic mellitus	8 (15.7)	9 (15.5)	1
Hypertension	15 (29.4)	17 (29.3)	1
Dyslipidemia	41 (80.4)	49 (84.5)	1
Coronary artery disease	2 (3.9)	2 (3.4)	1
Cerebrovascular disease	2 (3.9)	2 (3.4)	1
Liver function tests			
Total bilirubin: median (IQR), mg%	0.6 (0.4, 0.7)	0.6 (0.4, 0.8)	0.715
AST: median (IQR), U/L	31 (23, 41.2)	29 (24, 39)	0.606
ALT: median (IQR), U/L	40 (23.8, 59.5)	36 (27, 61)	0.760
ALP: median (IQR), U/L	79.5 (71.8, 100)	85 (64, 99)	0.758
Albumin: mean (SD), g%	4.6 (0.2)	4.6 (0.3)	0.628
Laboratory results			
Hematocrit: mean (SD), %	41.9 (3.6)	42.4 (3.8)	0.452
Platelet: mean (SD), × 10^3^/µL	274.6 (49)	266.2 (63.2)	0.421
Creatinine: median (IQR), mg%	0.7 (0.6, 0.9)	0.8 (0.6, 0.9)	0.724
HOMA-IR: median (IQR), mg/dL	2.6 (1.8, 3.8)	2.6 (1.4, 4.1)	0.455
HbA1c: median (IQR), %	5.7 (5.4, 6)	5.7 (5.5, 6.2)	0.668
LDL: median (IQR), mg%	121.3 (94.6, 142.1)	115.7 (100.5, 152)	0.797
FIBROSCAN data			
CAP: mean (SD), dB/m	292 (48)	288.1 (52.1)	0.668
Liver stiffness: median (IQR), kPa	5.6 (4.8, 7.3)	5.8 (5.1, 7.1)	0.407
Bioelectrical impedance analysis data			
Fat mass: median (IQR), kg	24.6 (19.9, 31)	24.6 (20, 30.3)	0.758
Percentage of fat: mean (SD), %	36.4 (9.2)	35.4 (8.1)	0.523
Muscle mass: median (IQR), kg	39.6 (36.7, 49.2)	40.7 (36.2, 51.4)	0.826
Skeletal muscle mass: median (IQR), kg	22.6 (21.4, 26.3)	23.4 (21.1, 28.4)	0.574

ALP, alkaline phosphatase; ALT, alanine aminotransferase; AST, aspartate aminotransferase; BMI, body mass index; CAP, controlled attenuation parameter; dB/m, decibels per meter; HbA1c, Hemoglobin A1c; HOMAR-IR, Homeostatic Model Assessment for Insulin Resistance; IQR, interquartile range; kPa, kilopascal; LDL, low-density lipoprotein; SD, standard deviation.

### Clinical outcomes: intention-to-treat analysis

The outcomes of the study from the intention-to-treat analysis are shown in Table 2. After a completed 24-week follow-up, patients in both intervention and control groups experienced a significant reduction in weight, BMI, ALT level, liver steatosis by CAP measurement, and fat mass by BIA compared to their respective levels at baseline (within group analysis). Interestingly, the significant improvement in liver stiffness (− 0.4, IQR: − 1.4 to 0.2, *P* = 0.008) and HGS (+ 0.7, IQR: − 0.7 to 3, *P* = 0.014) were observed only in the intervention group, not in the control group. While the skeletal muscle masses were significantly decreased in the control group but still maintained in the intervention group. Nonetheless, for the comparisons of outcomes at week 24 between the intervention and control groups, those differences in results were not statistically significant.

**Table 2:** Outcomes between intervention group and control group (Intention-to-treat analysis).

Variables	Control group (n = 61)	*P* value time	Intervention group (n = 61)	*P* value time	*P* value between group
Anthropometric measurement					
Weight: median (IQR), kg	70.3 (63.4, 78)	–	69 (61, 78.9)	–	0.854
Weight change: median (IQR), kg	− 1 (− 3.2, 0)	< 0.001	− 1.3 (− 3.1, 0)	< 0.001	0.599
Percentage weight change: median (IQR), %	− 1.3 (− 4.2, 0)	< 0.001	− 1.9 (− 4.2, 0)	< 0.001	0.525
BMI: mean (SD), kg	27.1 (3.9)	–	27.2 (4.1)	–	0.909
BMI change: median (IQR), kg/m^2^	− 0.4 (− 1.1, 0)	< 0.001	− 0.5 (− 1.1, 0)	< 0.001	0.530
Waist circumference: mean (SD), cm	91.4 (9.4)	–	90.9 (10)	–	0.764
Waist circumference change: median (IQR), cm	− 2 (− 3.5, 0)	< 0.001	− 1.5 (− 3, 0)	< 0.001	0.768
HGS change: median (IQR), kg	0.3 (− 0.9, 2.7)	0.092	0.7 (− 0.7, 3)	0.014	0.693
Laboratory results					
ALT: median (IQR), U/L	26 (20, 44)	–	31 (19, 53)	–	0.343
ALT change: median (IQR), U/L	− 9 (− 17.8, − 0.8)	< 0.001	− 4 (− 17, 2)	0.006	0.189
HOMA-IR: median (IQR), mg/dL	2.3 (1.4, 3.8)	–	2.3 (1.4, 3.3)	–	0.502
HOMA-IR change: median (IQR), mg/dL	− 0.1 (− 1, 0.6)	0.240	− 0.1 (− 0.9, 0.5)	0.102	0.804
FIBROSCAN data					
CAP: mean (SD), dB/m	267 (49.1)	–	267.9 (56.1)	–	0.925
CAP change: mean (SD), dB/m	− 25.1 (41.9)	< 0.001	− 19.9 (43.9)	< 0.001	0.506
Percentage CAP change: mean (SD), %	− 7.8 (14.4)	< 0.001	− 6.2 (15.4)	< 0.001	0.557
Liver stiffness: median (IQR), kPa	5.4 (4.6, 6.8)	–	5.4 (4.2, 6.4)	–	0.690
Liver stiffness change: median (IQR), kPa	− 0.1 (− 1.5, 1)	0.639	− 0.4 (− 1.4, 0.2)	0.008	0.225
Bioelectrical impedance analysis data					
Fat mass: mean (SD), kg	24.3 (18.9, 29.9)	–	22.9 (18.5, 28.4)	–	0.628
Fat mass change: mean (SD), kg	− 0.8 (2.1)	0.004	− 1.3 (2.3)	< 0.001	0.297
Percentage of fat mass: mean (SD), %	35.9 (9.4)	–	34.4 (8.4)	–	0.368
Percentage of fat mass change: mean (SD), %	− 0.5 (1.9)	0.040	− 1 (2.1)	< 0.001	0.213
Skeletal muscle mass: median (IQR), kg	22.6 (21.2, 26.7)	–	23.1 (21.35, 27.65)	–	0.404
Skeletal muscle mass change: median (IQR), kg	− 0.30 (0.9)	0.007	− 0.16 (0.9)	0.168	0.403
Physical activity					
Moderate exercise activity, min/week	40 (0, 150)	–	60 (0, 150)	–	0.539
Vigorous exercise activity, min/week	0	–	0	–	0.666
Any exercise activity, min/week	80 (0, 180)	–	120 (0, 225)	–	0.283

ALP, alkaline phosphatase; ALT, alanine aminotransferase; AST, aspartate aminotransferase; BMI, body mass index; CAP, controlled attenuation parameter; dB/m, decibels per meter; HbA1c, Hemoglobin A1c; HOMAR-IR, Homeostatic Model Assessment for Insulin Resistance; IQR, interquartile range; kPa, kilopascal; LDL, low-density lipoprotein; SD, standard deviation.

### Clinical outcomes: per-protocol analysis

In the per-protocol analysis, 2 patients in the control group and 4 patients in the intervention group were unable to come for their week 24 visit within the allowed timeframe (± 4 weeks) and were thus excluded. An additional 10 patients were also excluded: 4 in the control group due to the occurrence of unexpected event(s) that limited LSI activity or interfered with outcome measurements during the study period (minor stroke 1, indicated for hormonal therapy 1, herniated disc 1, and coronavirus disease 2019 (COVID-19) infection 1), and 6 in the intervention group (ankle injury 2, underwent knee surgery 1, severe knee pain limiting physical activity 1, indicated for corticosteroids treatment 1, and accidentally revealed the randomization arm to the investigator 1). Finally, a total of 106 patients (55 in the control group and 51 in the intervention group) were included in the per-protocol analysis.

The results of the per-protocol analysis are presented in Table 3. Both groups experienced significant weight reduction compared to baseline, with a greater degree of weight loss seen in the intervention group than in the control group but not statistically significant when compared between groups (− 2%, IQR: − 4.9, − 0.2 vs. − 1.6%, IQR: − 4.4, − 0.1, *P* = 0.407).

**Table 3:** Outcomes between intervention group and control group (Per-protocol analysis).

Variables	Control group (n = 55)	*P* value time	Intervention group (n = 51)	*P* value time	*P* value between group
Anthropometric measurement					
Weight: median (IQR), kg	70.3 (63.5, 78.5)	–	66.4 (60.5, 74.6)	–	0.316
Weight change: median (IQR), Kg	− 1.1 (− 3.4, − 0.1)	< 0.001	− 1.5 (− 3.1, − 0.1)	< 0.001	0.511
Percentage weight change: median (IQR), %	− 1.6 (− 4.4, − 0.1)	< 0.001	− 2 (− 4.9, − 0.2)	< 0.001	0.407
BMI: mean (SD), kg	26.4 (24.4, 28.8)	–	26 (23.6, 28.6)	–	0.481
BMI change: median (IQR), kg/m^2^	− 0.4 (− 1.2, 0)	< 0.001	− 0.6 (− 1.3, 0)	< 0.001	0.416
Waist circumference: mean (SD), cm	91.6 (9.8)	–	89.3 (9.5)	–	0.225
Waist circumference change: median (IQR), cm	− 2 (− 4.1, 0)	< 0.001	− 2 (− 4, − 1)	< 0.001	0.990
HGS change: median (IQR), kg	0.3 (− 0.8, 2.7)	0.089	0.3 (− 0.8, 2.7)	0.015	0.741
Laboratory results					
ALT: median (IQR), U/L	25 (20, 39.5)	–	28 (19, 45)	–	0.783
ALT change: median (IQR), U/L	− 9 (− 16, − 1)	< 0.001	− 5 (− 19.5, 1)	0.002	0.705
HOMA-IR: median (IQR), mg/dL	2.3 (1.4, 3.7)	–	2.1 (1.4, 2.9)	–	0.184
HOMA-IR change: median (IQR), mg/dL	− 0.2 (− 1, 0.5)	0.167	− 0.3 (− 1.3, 0.5)	0.047	0.578
FIBROSCAN data					
CAP: mean (SD), dB/m	267.7 (51)	–	261.1 (55.8)	–	0.526
CAP change: mean (SD), dB/m	− 27.5 (42.9)	< 0.001	− 23.6 (45.9)	< 0.001	0.655
Percentage CAP change: mean (SD), %	− 8.6 (14.7)	< 0.001	− 7.4 (16.2)	< 0.001	0.708
Liver stiffness: median (IQR), kPa	5.4 (4.6, 6.9)	–	5.4 (4.2, 6.3)	–	0.465
Liver stiffness change: mean (IQR), kPa	0.1 (2.4)	0.904	− 0.7 (1.8)	0.003	0.035
Bioelectrical impedance analysis data					
Fat mass: mean (SD), kg	25.9 (18.8, 29.8)	–	21.9 (18, 27.5)	–	0.270
Fat mass change: mean (SD), kg	− 1 (2.1)	0.002	− 1.6 (2.2)	< 0.001	0.187
Percentage of fat mass: mean (SD), %	35.7 (9.6)	–	34.1 (8.6)	–	0.354
Percentage of fat mass change: mean (SD), %	− 0.6 (1.9)	0.027	− 1.2 (2.1)	< 0.001	0.172
Skeletal muscle mass: median (IQR), kg	22.5 (21.2, 27)	–	22.8 (21.3, 27)	–	0.788
Skeletal muscle mass change: median (IQR), kg	− 0.4 (0.8)	0.003	− 0.3 (0.8)	0.005	0.809
Physical activity					
Moderate exercise activity, min/week	60 (0, 162.5)	–	90 (0, 155)	–	0.378
Vigorous exercise activity, min/week	0	–	0 (0, 30)	–	0.281
Any exercise activity, min/week	80 (0, 180)	–	120 (40, 255)	–	0.120

ALP, alkaline phosphatase; ALT, alanine aminotransferase; AST, aspartate aminotransferase; BMI, body mass index; CAP, controlled attenuation parameter; dB/m, decibels per meter; HbA1c, Hemoglobin A1c; HOMAR-IR, Homeostatic Model Assessment for Insulin Resistance; IQR, interquartile range; kPa, kilopascal; LDL, low-density lipoprotein; SD, standard deviation.

**Figure 2:**
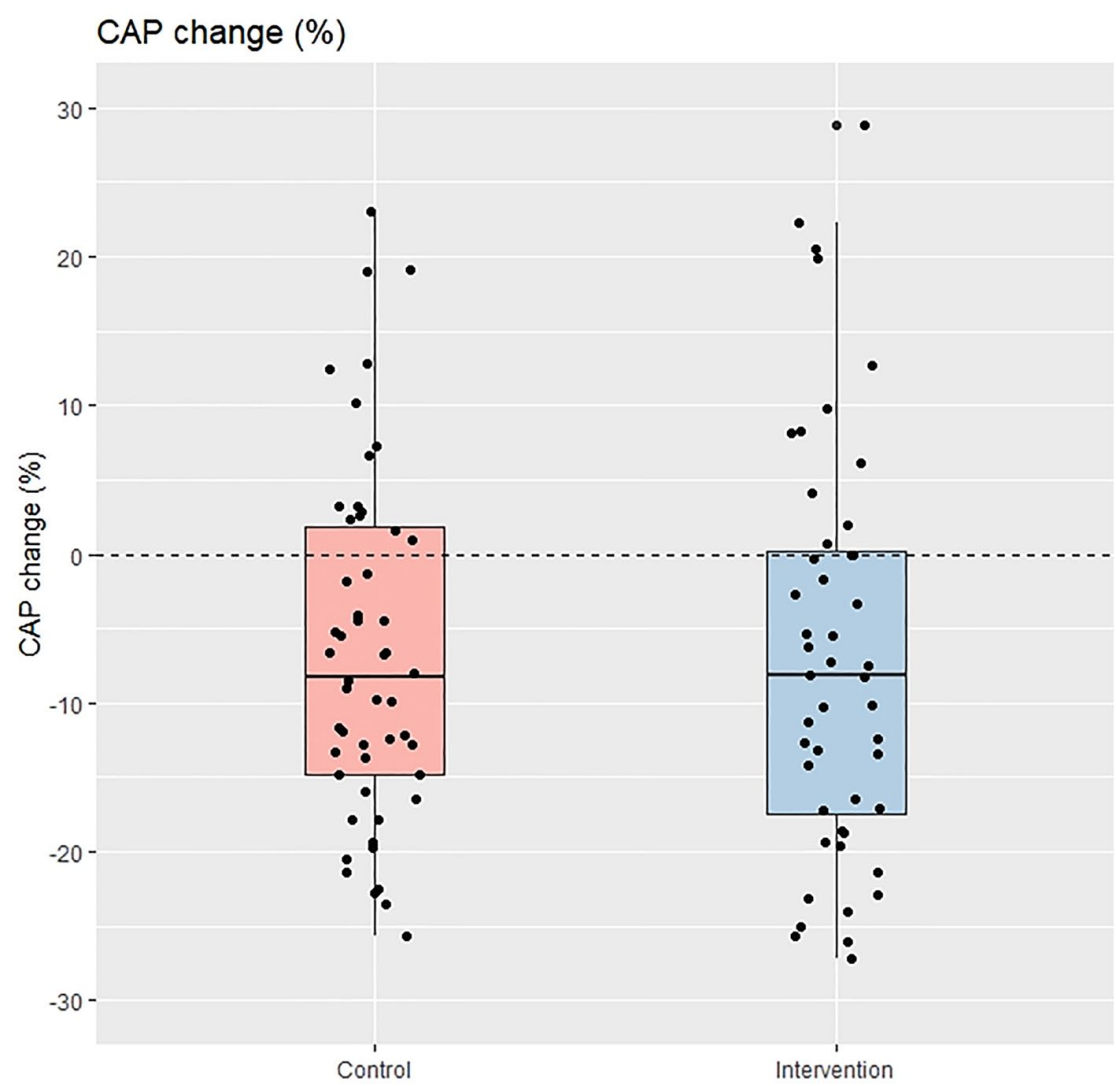
Percentage of CAP change between control group and intervention group (per protocol analysis).

**Figure 3:**
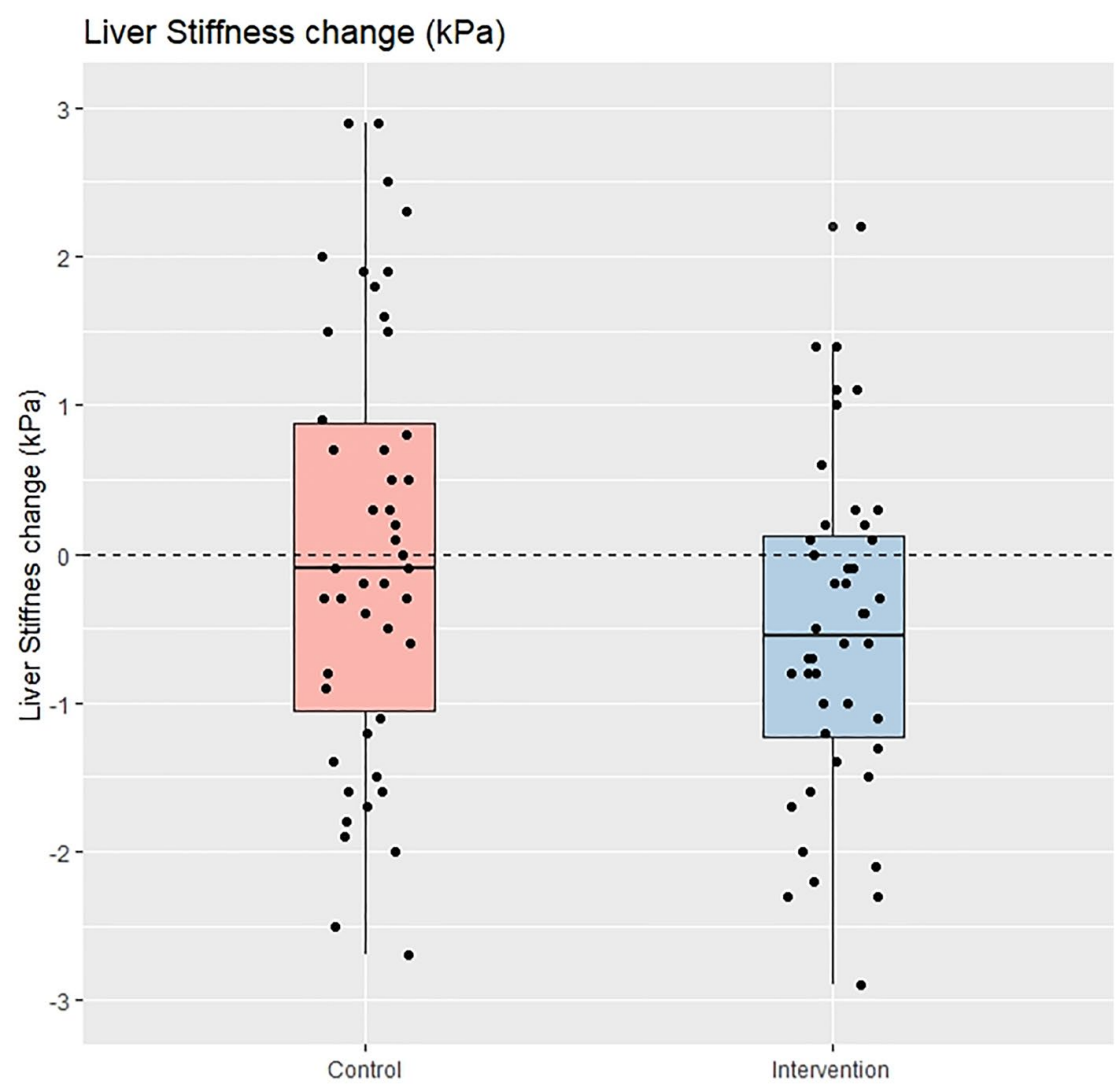
Liver stiffness change between control group and intervention group (per protocol analysis).

The degree of liver steatosis and ALT levels also decreased significantly compared to baseline in both groups but were not significantly different when analyzed for the between-group comparison. Notably, the patients in the intervention group experienced a significant reduction in liver stiffness (− 0.7 ± 1.8 kPa, *P* = 0.003) at 24-week, while the patients in the control group did not (± 0.1 + 2.4 kPa, *P* = 0.904), and this difference was also statistically significant after the between-group comparison was carried out (*P* = 0.035). Figures 2 and 3 depict the boxplot and the distribution of %CAP change and change in liver stiffness between the intervention and control groups.

Patients in the intervention group also had significantly better HOMA-IR and HGS strength at week 24 compared to baseline, while the same results were not observed in the control group. However, the differences in these results were not statistically significant for the between group comparisons. Similarly, patients in the intervention group reported more weekly exercise time than those in the control group, but the difference was not statistically significant (80, IQR: 0, 180 vs. 120, IQR: 40, 255) min per week, respectively (*P* = 0.12).

## Discussion

Weight reduction through LSI is the current mainstay treatment for MASLD or MAFLD. The results from our study showed that smartphone-assisted LSI via the LINE official account was associated with a trend towards a greater reduction in weight, BMI, body fat mass measured by BIA, and HOMA-IR in patients with MASLD compared with SOC. And more importantly, a significantly greater reduction in liver stiffness was observed in the per-protocol analysis.

The baseline characteristics of the MASLD patients in this study are similar to those using NAFLD criteria in a recent randomized trial evaluating the effect of different types of exercise conducted in Thailand at another institute in terms of female preponderance, mean BMI, as well as baseline CAP and body fat mass^25^. Although the percentage of patients with diabetes in our study was 13.9%, which was lower than the 30% observed in prior clinical trials evaluating the effect of web-based or application-based weight loss promotion in NAFLD patients^26,27^, it was comparable to the 17% observed in a recent study of Korean patients with MAFLD^28^.

Nowadays, technology and smartphones are omnipresent in people's daily lives around the world. While no effective therapeutic medications have been approved for patients with MASLD, the use of web-based or smartphone-assisted LSI to improve patient outcomes is intriguing. There were prior studies evaluating this approach in patients with NAFLD; Axley et al. conducted a randomized controlled trial in the United States, where they used text messaging to provide education on LSI or SOC. The study found that patients in the text messaging group achieved a higher degree of weight loss compared to those in the control group. However, there was no significant difference in ALT levels between the two groups^27^. Mazzotti et al. compared the web-based program for the education of LSI to face-to-face group education in a randomized trial involving patients with NAFLD and demonstrated that the web-based intervention was at least as effective as group-based face-to-face intervention in terms of weight and ALT level reduction^26^. Recently, Lim et al. published a randomized study evaluating the nBuddy mobile application to track diet and physical activity and induce behavioral changes to achieve optimal weight in patients with NAFLD in Singapore. In comparison to a single face-to-face session with a trained nurse, patients in the mobile application group experienced a significantly greater reduction in weight, ALT, and waist circumference^29^.

In this study, we were also interested in the use of smartphones to assist MASLD patients with lifestyle modification. Considering both the general population and patients with MASLD in Thailand, the majority of whom have smartphones, but accessing a website or downloading and utilizing an application designed specifically for their MASLD status may be troublesome due to the additional steps required beyond their routine smartphone use. The LINE application, on the other hand, is the most commonly used chat and messaging application among Thais and is popular in many Asian countries. The application has the function of LINE official account to broadcast messages, photos, and videos to the followers. This LINE official account is regarded as more pragmatic and, thus, has been selected as a tool to assist LSI in our study. In the intervention group, we utilized LINE official account for both knowledge delivery and reminders to perform LSI every 3–7 days, whereas in the control group, there was no active broadcast of any information. Nonetheless, patients in both groups received the same counseling regarding MASLD by the same hepatologist (AK) on the first day of enrollment.

The results of the present study showed that the patients in both groups experienced weight loss, waist circumference, and ALT reduction significantly. However, only 18% of the control group and 21.3% of the intervention group achieved ≥ 5% weight reduction at 24 weeks (*P* = 0.82). With regards to the MASLD-specific outcomes, an improvement in the degree of liver steatosis measured by CAP was observed in both groups, but the improvement in liver stiffness was only seen in the intervention group in both intention-to-treat and per-protocol analyses. Overall, patients in the intervention group tended to have a greater degree of weight loss and fat mass reduction, improvement in liver stiffness, and better muscle mass and HGS than those in the control group, but the between group comparison’s significant levels were not reached. Only the liver stiffness improvement in the per-protocol analysis was significantly better in the intervention group (− 0.7 ± 1.8 kPa) than in the control group (+ 0.1 ± 2.4 kPa, *P* = 0.035).

In terms of weight loss, the results of our study are similar to the studies by Axley et al. and Lim et al. that those who were assigned to the smartphone-assisted group achieved a greater degree of weight loss than in the usual care group^27,29^. Although it showed only a trend without a statistically significant difference in our study, this might reflect the utility of smartphones in aiding weight reduction in fatty liver patients. However, the details of smartphone use in each study varied; in the Axley et al. and Lim et al. studies, they were more likely to be two-way as the patients needed to input their data onto the application or reply to the messages^27,29^. While our study was designed to be more pragmatic in real-life practice, it was rather one-way, as the patients only needed to read the content that was sent to them and get a reminder to perform LSI on their own. Therefore, the magnitude of the effect of intervention in our study may be lower than in prior studies.

In light of liver-specific outcomes, our study is the first to evaluate the degree of fibrosis and liver stiffness among the studies of web-based or smartphone-based LSI in patients with MAFLD/MASLD. Most of the prior studies assessed only anthropometric measurements and serum biochemistry data. Interestingly, while the degree of CAP reduction and weight loss were not significantly different between the intervention and the control groups, liver stiffness improvement was demonstrated in patients in the intervention group. This finding could be explained by the higher exercise time per week at the end of the study, the greater degree of fat mass loss and improvement in HOMA-IR observed (albeit nonsignificant) in the intervention group, as well as the lower degree of muscle mass loss and increased HGS observed in the intervention group than in the control group. Exercise has been shown to benefit liver fibrosis in ways beyond weight loss^23,30^. Nevertheless, the specific mechanisms responsible for these possible advantages have yet to be established. While sarcopenia, on the other hand, is associated with an increased risk of significant fibrosis in fatty liver patients^31^. However, as the significant improvement in liver stiffness was shown only in the per-protocol analysis, further studies are needed to confirm this finding.

The virtues of the present study are as follows: First, this is a randomized controlled trial in which both patients and outcome assessors (a doctor who performed FIBROSCAN, BIA measurement, and follow-up with the patients) were blinded to treatment allocation. Despite it was a smartphone-assisted LSI study, the patients in both groups were added to different LINE official accounts which appeared to be almost identical to one another. And only one investigator (SA) knows the random sequence and is the administrator of the LINE official account of both groups. SA has no role in either patient management or outcome assessment. Thus, the study has a very low potential for bias. Furthermore, as mentioned earlier, our study is the first to demonstrate liver steatosis and fibrosis outcomes among RCTs using websites or smartphones in fatty liver patients. Previous studies reported changes in anthropometric measurements and liver biochemistry, but none reported liver-specific outcomes e.g., liver steatosis or fibrosis change^26,27,29^. Additionally, the tool used in this study is pragmatic in everyday use and at a low cost; it may be adopted to be used nationwide in Thailand and in many countries, and it would be easier to approach a larger number of MASLD patients than using a more sophisticated technology that required a higher literacy level of both patients and doctors to achieve the outcomes. And lastly, it might be useful in situations where frequent follow-up visits are difficult. As the present study was conducted during the COVID-19 pandemic, in which weight gain was commonly encountered and non-emergency hospital visits were considered to be hindered^32,33^, the patients in our study were still able to lose weight (median %weight change was − 1.6% (IQR: − 4.2,0) overall) at the end of the study.

We also acknowledge that our study has some limitations. FIBROSCAN was used to assess liver steatosis and fibrosis in this study, rather than the gold standard of liver biopsy, which carries the risk of rare but potentially life-threatening complications. However, among the noninvasive quantitative assessments of liver steatosis and fibrosis, FIBROSCAN is the most widely validated test with reliable accuracy^34^. And although we attempted to validate the adherence in LSI using GPAQ for physical activity, unfortunately, there was no standard questionnaire to assess intake for Thai food, and we were unable to ascertain whether all patients in the intervention group actually read our content. As we aimed to make this study as pragmatic as possible, these limitations are to be traded off. Additionally, we recognize that participants may engage in discussions among themselves; even if they were explicitly asked not to do so, we could not ensure that on the participants’ side, it was a completely blinded fashion. Our study evaluated physical activity and muscle mass, as well as muscle strength (in terms of HGS), but cardiovascular fitness was not assessed, which might be another limitation as it is renowned that cardiovascular mortality is the leading cause of death in patients with MASLD. Nonetheless, there were prior data suggesting that the higher HGS, which was observed more commonly in the intervention group in our study, was associated with a lower risk of developing cardiovascular events^35^. And our study was originally designed to evaluate liver steatosis and stiffness outcomes. Furthermore, the follow-up time in this study was only 6 months, the beneficial effect of this approach in patients with MASLD beyond 6 months is yet to be explored. And lastly, MASLD patients who were included in the study had median liver stiffness 5.6–5.8 kPa, indicating that most participants had fibrosis stage 0–1, which were at low-risk of developing long-term liver-related complications. Further studies focusing on at least fibrosis stage 2 might demonstrate more clinically meaningful results.

In conclusion, our study showed that reminding, encouraging LSI, and delivering MASLD information via a social media application (LINE official account) to patients with MASLD demonstrated a significantly better outcome of liver stiffness than SOC, and the outcomes regarding weight loss, body composition change, as well as HGS and HOMA-IR tended to be better than SOC alone, although the comparisons between groups were not statistically significant. While awaiting for the effective therapeutic medications to be approved, this smartphone-assisted approach can be considered to enhance LSI in MASLD patients for better outcomes.

### Supplementary Information

Supplementary Figure 1.

## Data Availability

The data that support the findings of this study are available on request from the corresponding author.

## Abbreviations

AASLD: The American Association for the Study of Liver Diseases
ALD: Alcoholic liver disease
ALT: Alanine aminotransferase
APASL: The Asian Pacific Association for the Study of the Liver
BIA: Bioelectrical impedance analysis
BMI: Body mass index
BUN: Blood urea nitrogen
CAP: Controlled attenuation parameter
CBC: Complete blood count
Cr: Creatinine
EASL: The European Association for the Study of the Liver
FBS: Fasting blood sugar
FLD: Fatty liver disease
GGT: Gamma-glutamyl transferase
GPAQ: Global physical activity questionnaire
HbA1C: Hemoglobin A1C
HGS: Hand grip strength
HOMA-IR: Homeostasis model assessment parameter of insulin resistance
IQR: Interquartile range
kPa: Kilopascals
LFT: Liver function test
LSI: Lifestyle intervention
LSM: Lifestyle modification
MASLD: Metabolic dysfunction-associated steatotic liver disease
NAFLD: Non-alcoholic fatty liver disease
RCPT: Royal College of Physician of Thailand
SD: Standard deviation
SOC: Standard of care
TCTR: Thai Clinical Trials Registry
VCTE: Vibration-controlled transient elastography
