# Supplementary material for: Effect of smartphone-assisted lifestyle intervention in MASLD patients: a randomized controlled trial
Source: Sci Rep. 2024 Jun 17;14:13961. doi: 10.1038/s41598-024-64988-4 (PMC11183044; doi:10.1038/s41598-024-64988-4)

**Research Article**

**Effect of Smartphone-assisted Lifestyle Intervention in MASLD Patients: A Randomized Controlled Trial.**

Apichat Kaewdech^1,†^, MD, Suraphon Assawasuwannakit^1,2, †^, MD, Chaitong Churuangsuk^3^, MD, PhD, Naichaya Chamroonkul^1^, MD, Pimsiri Sripongpun^1^, MD

^1^Gastroenterology and Hepatology Unit, Division of Internal Medicine, Faculty of Medicine, Prince of Songkla University, Songkhla, Thailand

^2^Department of Medicine, Panyananthaphikkhu Chonprathan Medical Center, Srinakharinwirot University, Nonthaburi, Thailand

^3^Clinical Nutrition and Obesity Medicine unit, Division of Internal Medicine, Faculty of Medicine, Prince of Songkla University, Songkhla, Thailand

^†^Authors contributed equally to this work and shared co-first authors.

**Supplementary Figure 1:** Examples of a screenshot and the content sent via LINE official account to the intervention group.


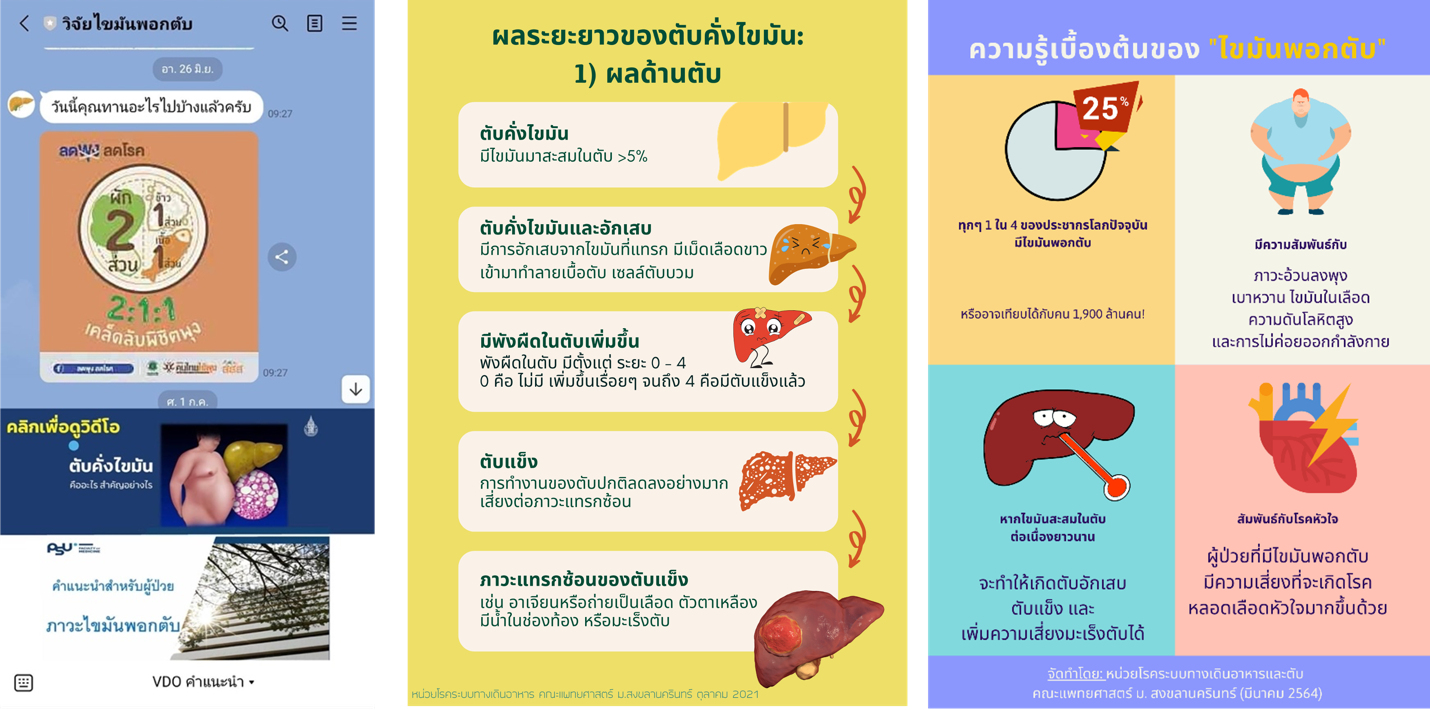

Supplement: Supplementary file 1 — Supplementary Figure 1. [file 41598_2024_64988_MOESM1_ESM.docx]
